# Temperature-Related Bioclimatic Variables Play a Greater Role in the Spatial Distribution of Bumblebee Species in Northern Pakistan

**DOI:** 10.3390/insects16010001

**Published:** 2024-12-24

**Authors:** Muhammad Naeem, Arzoo Rani, Weiyao Lyu, Huaibo Zhao, Maryam Riasat, Saail Abbas, Sabir Hussain, Nawaz Haider Bashir, Qiang Li, Huanhuan Chen

**Affiliations:** 1 College of Biological Resource and Food Engineering, Qujing Normal University, Qujing 655011, China; naeem@mail.qjnu.edu.cn (M.N.); nawazhaider@caf.ac.cn (N.H.B.); 2 Department of Zoology, Faculty of Engineering and Applied Sciences, Riphah International University, Faisalabad Campus, Faisalabad 38000, Pakistan; 3 Qilin District Livestock Improvement Station, Qujing 655000, China; 4 Insect Pest Management Program, Institute of Plant & Environmental Protection, National Agricultural Research Centre, Islamabad 45500, Pakistan; shussain7043@parc.gov.pk

**Keywords:** geographic information system, species distribution modeling, pollinator, spatial distribution, conservation

## Abstract

Bumblebee species are vital wild pollinators, providing essential pollination services for various crops, fruits, and vegetables. However, their biodiversity is vulnerable to decline due to climate change, particularly in regions like northern Pakistan. Despite this, no research has yet been conducted on the distribution patterns of bumblebee species in this region. The current study aimed to model the spatial distribution of three important bumblebee species: *Bombus haemorrhoidalis*, *B. rufofasciatus*, and *B. subtypicus* in northern Pakistan. Habitat suitability and the contribution of bioclimatic variables to the spatial distribution of species were assessed using the MaxEnt approach. Current and future bioclimatic variables, along with presence-only records of three bumblebee species, were incorporated into the species distribution model. The results indicated that nearly 96% of the area (43 out of 45 cities in northern Pakistan) showed habitat suitability for all three species in the current scenario. Among these 43 cities, five exhibited a 100% overlap in suitable areas for the three species. However, this overlap area is expected to decrease in the future, particularly by the middle of the 21st century, highlighting these regions as prime candidates for conservation. In terms of bioclimatic factors influencing spatial distribution, the study found that temperature-related variables played a more significant role than precipitation-related ones in current and future scenarios. Specifically, bio3 (isothermality) contributed 48% to *B. haemorrhoidalis* and 43% to *B. rufofasciatus*, while bio2 (mean diurnal range) was the most influential factor for *B. subtypicus*. Temperature-related variables accounted for more than 80%, 69.4%, and 78.3% of the spatial variation in *B. haemorrhoidalis*, *B. rufofasciatus*, and *B. subtypicus*, respectively. This study demonstrates the critical influence of temperature on the spatial distribution of bumblebee species in northern Pakistan, underscoring the need for climate-focused conservation strategies to protect these important wild pollinators.

## Simple Summary

Over 250 bumblebee species have been reported worldwide, making them some of the most important wild pollinators, providing essential pollination services for many wild flowers and crops, including fruits and vegetables. However, their populations are declining due to various factors, including climate change. In Pakistan, only the northern areas provide more suitable habitats for these bumblebee species, but their populations are under threat in this region due to global warming and climate change. In this study, we identified the most suitable areas for bumblebee conservation and examined the contribution of bioclimatic factors to their spatial distribution.

## 1. Introduction

Bumblebees, belonging to the genus *Bombus* (a group of large social insects), are among the most effective pollinators, providing essential pollination services that support both biodiversity and agricultural productivity worldwide [1,2]. They contribute to the pollination of wild plants and agricultural crops alike [3]. In Pakistan, most bumblebee species are concentrated in the northern regions, where the abundance of flowers and favorable climate provide suitable habitats [4]. In these mountainous regions, bumblebees contribute to diverse ecological systems. However, local bioclimatic factors significantly influence the distribution range of these species, highlighting the importance of understanding how these factors shape bumblebee habitats [5].

Temperature, precipitation, humidity, and seasonality are all bioclimatic and environmental factors that shape species distributions by defining the ecological conditions suitable for their survival [6]. Bumblebee species are particularly sensitive to these bioclimatic factors, and their distribution is closely linked to these environmental conditions [7]. Therefore, studying bioclimatic factors is essential for understanding the ecological range and habitat preferences of bumblebee species within a particular region.

Previous studies have also focused on the distribution patterns of bumblebee species in relation to bioclimatic factors. For example, in the Tibetan region of China, various groups of bumblebee species exhibit distinct distribution patterns across different areas, with each group evolving in association with specific bioclimatic factors [8]. Bumblebee species establish their ecological boundaries by responding to changes in temperature and precipitation [9]. These distribution patterns and responses to bioclimatic conditions are essential to study at a local scale for each species [10,11]. Despite evidence that bioclimatic factors play a key role in the distribution patterns of bumblebee species in different regions, our knowledge remains limited regarding bumblebee species in northern Pakistan. Although over ten bumblebee species have been reported in Pakistan, three species are particularly abundant: *Bombus haemorrhoidalis* Smith, 1852; *B. rufofaciatus* Smith, 1852; and *B. subtypicus* (Skorikov, 1914) [12]. However, their local distribution patterns remain largely unknown.

Furthermore, global climate change is a major driver of biodiversity loss [13]. In various regions worldwide, climate change has led to shifts in bumblebee ranges [14], and widespread declines in bumblebee species have also been documented [15]. In North America and Europe, long-term observation data spanning approximately 110 years have been used to assess the movement or range shift in bumblebee species due to climate change, with range losses observed in southern regions [10]. Similarly, a climate change study conducted in China showed that more than 70% of bumblebee species are at risk. Furthermore, climate change increases the risk of alien species invasion [7,16,17]. At the local scale, such studies are important for regions where no previous research has been conducted.

The limited research on the role of bioclimatic factors in shaping the distribution of bumblebee species in the northern landscapes of Pakistan highlights a significant gap in understanding how these factors influence bumblebee distribution. Although studies from other regions provide a valuable foundation [16,17,18], the unique climatic and topographic conditions of northern Pakistan may lead to distinct distribution patterns or ecological requirements for these species, underscoring the need for focused investigation. The aim of this study is to assess the influence of current bioclimatic factors on the spatial distribution of the three key bumblebee species, *B. haemorrhoidalis*, *B. rufofasciatus*, and *B. subtypicus*, in northern Pakistan. The objectives are to (1) map the spatial distribution of these species in northern Pakistan, (2) identify the most influential factors shaping the specific distribution patterns of these bumblebee species in this region, and (3) identify the area of prime conservation potential for these three bumblebee species in northern Pakistan.

## 2. Materials and Methods

### 2.1. Site Selection and Data Gathering

**Figure 1:**
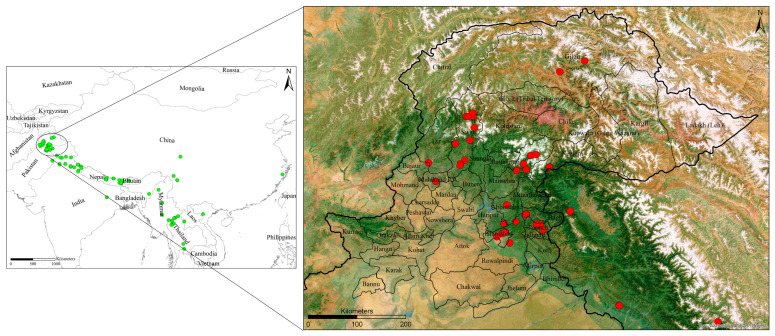
Bumblebees’ collection sites (green and red bolls) in north part of Pakistan and its surrounding regions.

To assess the local distribution patterns and the contribution of bioclimatic factors, we gathered records for three important bumblebee species: *B. haemorrhoidalis*, *B. rufofasciatus*, and *B. subtypicus*. The data on the geographic coordinates of these species were obtained from the Global Biodiversity Information Facility (GBIF, http://www.gbif.org) (access on 5 November 2024) in November 2024. For the local data we also focused on literature [12,19,20,21,22,23]. The total number of collection sites was 272. All collection sites were separated by a distance of more than 10 km from each other; therefore, there was no need to apply spatial filtering. The study area encompasses the northern region of Pakistan, which includes 45 cities across Azad Kashmir, FATA, Khyber Pakhtunkhwa, Gilgit-Baltistan, and the Punjab provinces. The total area selected for the study of distribution patterns was 178,372 km^2^. The distribution sites of these three bumblebee species were located within this study region (Figure 1).

### 2.2. Modeling Procedure

For the assessment of spatial distribution modeling and to determine the contribution of the most important bioclimatic variables, we utilized 19 bioclimatic layers from www.worldclim.org (access on 15 October 2024) with a spatial resolution of approximately 1 km^2^ (Table 1). To reduce multicollinearity among these bioclimatic variables, we used the Species Distribution Modeling Toolbox of ArcGIS v10.0 and only included those bioclimatic variables that showed Pearson correlation coefficient values less than 0.8 [16,24,25].

To assess the future distribution of these three bumblebee species, we used bioclimatic data for two future time periods (2021–2040 and 2041–2060). The future climatic projections were based on four Shared Socio-economic Pathways (SSPs); however, we focused on two scenarios from the global climate model (GCM) ACCESS-CM2: SSP1 and SSP4, for both time periods (2021–2040 and 2041–2060). These scenarios represent contrasting radiative forcing levels, with SSP1 indicating low radiative forcing (2.6 W/m^2^) and SSP4 indicating high radiative forcing (8.5 W/m^2^), corresponding to minimum and maximum levels of greenhouse gas emissions and socioeconomic changes, respectively.

The maximum entropy (MaxEnt) model was selected to assess the spatial distribution of the three most important bumblebee species in the northern areas of Pakistan (Figure 1). The default feature types and regularization settings in MaxEnt were used. Since we only have presence data, MaxEnt is an ideal choice as it performs well with such data by applying the principles of maximum entropy to assess the potential distribution of species using bioclimatic variables [25]. MaxEnt used a total of 10,000 background points, which is the default setting in MaxEnt and is considered a standard practice for ensuring robustness and producing precise modeling outputs for species. The bioclimatic variables, which are temperature- and precipitation-related factors, are known to have a significant impact on bumblebee distribution [26]. All 12 bioclimatic raster layers (Table 1) for the current and future scenarios were converted into ASCII format using ArcGIS v10.0 for use in MaxEnt v3.3.3k software for species distribution modeling. Similarly, all occurrence records of three bumblebee species were converted into CSV (comma-delimited) format for input into MaxEnt v3.3.3k. For MaxEnt, we created a bias file based on the occurrence records of the three species to guide background point selection and ensure a more accurate prediction of spatial distribution. This bias file was created using ArcGIS v10.0. We ran the MaxEnt model for all regions of Asia where bumblebee samples were collected for the current and future scenarios. After obtaining the species distribution output, the focus was narrowed to northern Pakistan [27].

The output of the MaxEnt model, based on the relationship between occurrence records and bioclimatic variables, is a logistic probability with values ranging from 0 (unsuitable habitat) to 1 (highly suitable habitat). The accuracy of the MaxEnt model was evaluated using AUC (Area Under the Curve) values from ROC (Receiver Operating Characteristic) curves and TSS (True Skill Statistics) values. AUC values range from 0 to 1, with higher values indicating better model accuracy, while TSS values range from −1 to 1, with values above 0.5 suggesting good model performance [25,28].

Using ArcGIS software, the predictive model outputs were processed and used to determine habitat suitability for bumblebees. This process allowed the application of spatial analysis to produce thematic maps depicting the predicted distribution patterns of bumblebees in the study area. This evaluation provides valuable insights into the relationships between bioclimatic factors and bumblebee distribution in the research region.

## 3. Results

### 3.1. Modeling Accuracy

For the accuracy of our analysis, AUC values for the training data ranged from 0.97 to 0.99, and similarly, AUC values for the test data ranged from 0.97 to 0.99 across all three bumblebee species: *B. haemorrhoidalis*, *B. rufofaciatus*, and *B. subtypicus*. The 10th percentile training presence logistic threshold values varied from 0.147 to 0.411, with fractional predicted areas ranging from 0.021 to 0.068. Additionally, TSS values ranged from 0.55 to 0.86, indicating robust model performance (Table 2).

### 3.2. Spatial Distribution of Bumblebee Species

**Figure 2:**
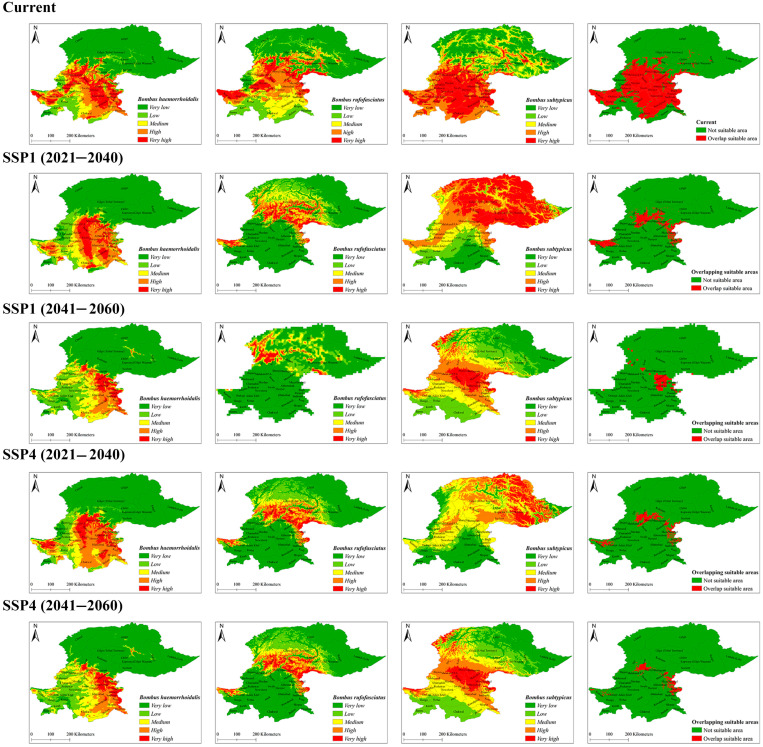
Spatial distribution of habitat suitability areas and overlap areas of the three most important bumblebee species in north part of Pakistan at current and future scenarios.

Distribution maps of current and future scenarios for all three species were converted into binary maps based on the 10th percentile logistic threshold values, and overlapping areas were identified. The overlapping area represents regions with the highest habitat suitability for all three species in northern Pakistan (Figure 2). Suitable habitats shared by all three species were found across 43 cities, with the percentage of suitable overlap area ranging from 0.04% to 100%. This overlap is predicted to decrease in the future (Table 3 and Figure 2).

In the current scenario, the five cities, Islamabad, Mardan, Poonch, Sudhnati, and Swabi, showed 100% overlap in suitable habitat areas for all three species. The areas of these five cities have been identified as the most important regions for implementing conservation strategies. Under the SSP1 scenario, the overlap is predicted to decrease substantially, with values ranging from 0% in Islamabad, Swabi, and Mardan to a maximum of 4.71% in Poonch and 6.25% in Sudhnati in the future (Table 3). Similarly, under the SSP4 scenario, the overlap in suitable areas is projected to decline further, ranging from 0% in Islamabad and Swabi to 32.09% in Poonch by 2060 (Table 2). These results indicate a substantial reduction in habitat overlap under future climate scenarios, with notable variations between cities (Table 3).

For the individual species, *B. haemorrhoidalis* had very high suitable areas in the 16 cities across northern Pakistan, with suitable area ranges between 44 km^2^ and 2671 km^2^ under the current scenario (Figure 2). These cities, listed in decreasing order of suitable area, include Swat, Dir, Kurram, Mansehra, Abbottabad, Muzaffarabad, Bagh, Kohistan, Shangla, Attock, Khyber, Battagram, Neelum, Kohat, Karak, and Mohmand (Table 4). For the future, this species is projected to have highly suitable areas in 12 to 14 cities. Further details about future prediction across different cities are present in Supplementary Materials (Tables S1–S12).

For *B. rufofasciatus*, very high suitability was found in 15 cities, with suitable areas ranging from 21 km^2^ to 4737 km^2^ (Figure 2 and Table 5). The largest areas of very high suitability for this species were in Swat, followed by Neelum, Kohistan, Mansehra, Khyber, Dir, Battagram, Chilas, Shangla, Kupwara (Gilgit Wazarat), Kargil, Attock, Gilgit (Tribal Territory), Chitral, and Gilgit (Table 5). For the future, this species is projected to have highly suitable areas in 13 to 18 cities under the SSP1 and SSP2 scenarios (see Supplementary Materials).

Similarly, *B. subtypicus* showed very high suitability in 14 cities, with areas ranging from 45 km^2^ to 4509 km^2^ (Figure 2). The highest areas of very high suitability for this species were in Mansehra, followed by Swat, Dir, Shangla, Kargil, Neelum, Kohistan, Battagram, Gilgit, Kupwara (Gilgit Wazarat), Chilas, Ladakh (Leh), Chitral, and Gilgit (Tribal Territory) (Table 6). The highest suitability for this species was limited to one to three cities under both SSP1 and SSP4 future scenarios (see Supplementary Materials).

**Figure 3:**
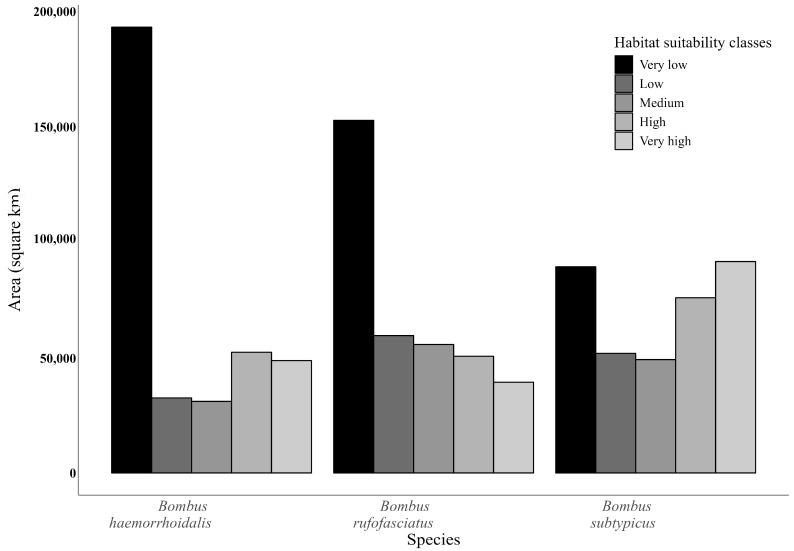
The distribution of area (km^2^) by different habitat suitability class of all three bumblebee species in north part of Pakistan.

Each of the three species, *B. haemorrhoidalis*, *B. rufofasciatus*, and *B. subtypicus*, exhibits habitat suitability distributed across five classes: very low, low, medium, high, and very high (Figure 3).

The area covered by each habitat suitability class varies among these species, reflecting differences in the extent and concentration of suitable habitats across the northern region of Pakistan (Figure 2). For *B. haemorrhoid––alis*, the largest area is covered by the very low suitability class, followed by decreasing extents in the high, very high, low, and medium suitability classes. In contrast, *B. rufofasciatus* occupies the largest area in the very low suitability class, followed by low, medium, high, and very high suitability classes (Figure 3). *B. subtypicus*, on the other hand, is predominantly found in the very high habitat suitability class, followed by very low, high, low, and medium suitability classes (Figure 3).

**Figure 4:**
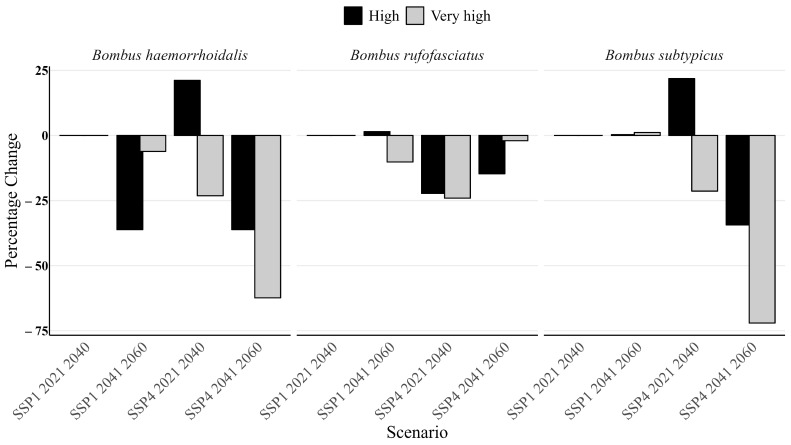
Percentage range changes in habitat suitability ranges in three bumblebee species during the future time periods in north part of Pakistan.

Under future climate scenarios (SSP1 and SSP4), a significant reduction in the high and very high habitat suitability classes was detected for all three bumblebee species. In *B. haemorrhoidalis*, the very high suitability habitat class showed a range reduction of up to 62% under the SSP4 scenario during the years 2041–2060 compared to current baseline values. The very high suitability range of *B. rufofasciatus* decreased by up to 24% under the SSP4 scenario for the period 2021–2040. Lastly, *B. subtypicus* exhibited the most substantial loss, with the very high suitability range declining by up to 72% under the SSP4 scenario during 2041–2060 (Figure 4).

### 3.3. Contribution of Most Important Variables in the Spatial Distribution of Most Important Bumblebee Species in Northern Areas of Pakistan

**Figure 5:**
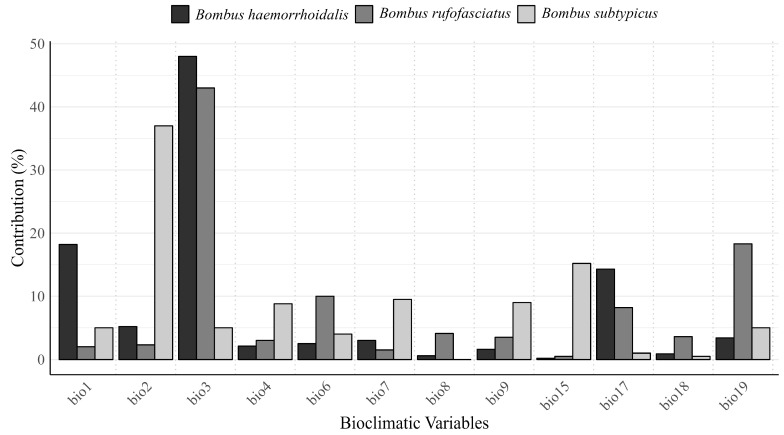
Contribution of bioclimatic factors in the spatial distribution of the three most important bumblebee species in north part of Pakistan.

Overall, temperature-related bioclimatic variables contributed more than precipitation-related variables to the spatial distribution of the three bumblebee species (Figure 5). For *B. haemorrhoidalis*, the temperature-related variable bio3 made the highest contribution (48%), followed by bio1 (18.2%). In the case of *B. rufofasciatus*, bio3 contributed the most (43%), while for *B. subtypicus*, bio2 had the highest contribution (37%). Temperature-related bioclimatic variables (bio1–bio11) contributed more than 80%, 69.4%, and 78.3% to the spatial distribution of *B. haemorrhoidalis*, *B. rufofasciatus*, and *B. subtypicus*, respectively. In contrast, precipitation-related bioclimatic variables contributed 18.8%, 30.6%, and 21.7% to the spatial distribution of *B. haemorrhoidalis*, *B. rufofasciatus*, and *B. subtypicus*, respectively (Figure 5). Furthermore, the future contribution of bioclimatic factors also showed that temperature-related variables played a larger role in the spatial distribution of the three bumblebee species across all future scenarios (Figure S1).

## 4. Discussion

Our findings indicate that temperature-related bioclimatic factors play a more significant role in the spatial distribution of three bumblebee species: *B. haemorrhoidalis*, *B. rufofasciatus*, and *B. subtypicus* (Figure 5). Temperature-related variables contributed up to 80% for *B. haemorrhoidalis*, followed by 65.3% for *B. rufofasciatus* and 65.1% for *B. subtypicus*. These results are consistent with previous studies emphasizing the critical role of temperature in shaping the spatial distribution of bumblebee species [29]. Notably, bio3 (isothermality) contributed over 50% to the spatial distribution of *B. haemorrhoidalis*, 43% for *B. rufofasciatus*, and bio2 contributed 37% to the distribution of *B. subtypicus*, underscoring that temperature-related variables are key determinants in shaping the geographic range of these species. These temperature-related factors are also considered the most influential in affecting the foraging and reproductive behavior of bumblebees [30].

Although precipitation-related variables also contribute to the spatial distribution of these three bumblebee species, their contribution is less compared to that of temperature-related factors (Figure 5). For example, the overall contribution of precipitation-related variables was 20%, 34.6%, and 34.9% for *B. haemorrhoidalis*, *B. rufofasciatus*, and *B. subtypicus*, respectively. This suggests that water availability is important, but secondary, in determining the distribution of these species in northern Pakistan (Figure 2). These findings highlight the importance of water content in regulating the habitat suitability for bumblebee species, as discussed by a previous study [31].

We identified overlap areas of high suitability for all three bumblebee species in the northern regions of Pakistan. Almost 96% of the area (43 out of 45 cities in northern Pakistan) showed habitat suitability for all three species. Among these 43 cities, five cities exhibited 100% overlap in suitable areas for the three species, indicating that these areas are of prime importance for conservation (Figure 2). Our findings also revealed that the three species have diverse ecological preferences. *B. haemorrhoidalis* and *B. rufofasciatus* were associated with a broader range of habitats, primarily in the “very low” suitability class, whereas *B. subtypicus* predominantly occupied areas within the “very high” suitability class (Figure 3).

The bumblebee species *B. haemorrhoidalis*, *B. rufofasciatus*, and *B. subtypicus* thrive in the climate of Northern Pakistan, which has the perfect temperature, humidity, and precipitation levels. Other regions may not have the same conditions [32]. The flora and habitat structures in Northern Pakistan are crucial for the foraging and nesting needs of these bumblebee species, which are not found in other regions [33].The higher altitudes and varied terrain of Northern Pakistan make it more suitable for these species compared to the flatter and hotter southern regions [34]. Each species has developed to take advantage of different ecological niches, resulting in differences in how they react to bioclimatic factors like temperature, precipitation, and seasonality [35].

MaxEnt achieved the best results compared to other modeling methods, which aligns with past research [36]. The findings of the research are crucial for developing successful conservation plans. By recognizing the important bioclimatic elements that impact the distribution of *B. haemorrhoidalis*, *B. rufofaciatus*, and *B. subtypicus*, we can better anticipate changes in bumblebee habitats and make appropriate preparations [26]. Conservationists can determine and prioritize important habitats for protection and restoration by studying how species are distributed in relation to temperature, precipitation, and elevation [37].

Future climate change scenarios indicate a substantial decline in habitat suitability for all three bumblebee species in northern Pakistan. The overlap areas of suitable habitats are also decreasing. For example, cities such as Islamabad, Mardan, and Swabi, which currently exhibit 100% overlap in suitable habitat areas, are predicted to experience a complete loss of overlap under future scenarios. Similarly, two other cities, Poonch and Sudhnati, that presently show a 100% overlap are projected to see a reduction to 4.71% and 6.25%, respectively (Table 2). This reduction is attributed to shifts in bioclimatic conditions in the future [38]. The decline in the very high suitability class for *B. haemorrhoidalis* and *B. subtypicus* by up to 62% and 72%, respectively, under the SSP4 scenario underscores the vulnerability of these species in northern Pakistan to warming temperatures and changing climatic patterns.

Our findings of this study are consistent with previous studies indicating that habitat suitability is critical to species distribution [30]. The results of this study enhance our understanding of the spatial distribution of these species and provide insight into their habitat requirements, which are essential for implementing effective conservation strategies [39,40]. The distinct habitat suitability patterns observed for each species suggest that conservation strategies should consider species-specific habitat preferences, particularly in regions where overlapping suitable habitats were identified and predicted to decline in the future in northern Pakistan.

## Figures and Tables

**Table 1:** Bioclimatic variables used to assess the spatial distribution of the three most important bumblebee species, *Bombus haemorrhoidalis*, *B. rufofasciatus*, and *B. subtypicus*, in north part of Pakistan. Bioclimatic variables with an asterisk (*) were included in our modeling because their Pearson’s correlation coefficients (r) were <0.8.

Sr. No.	Variable Names	Abbreviations
1	* Annual Mean Temperature	* bio1
2	* Mean Diurnal Range (Mean of monthly (Max Temp–Min Temp))	* bio2
3	* Isothermality (BIO2/BIO7) (×100)	* bio3
4	* Temperature Seasonality (Standard Deviation ×100)	* bio4
5	Max Temperature of Warmest Month	bio5
6	* Min Temperature of Coldest Month	* bio6
7	* Temperature Annual Range (BIO5-BIO6)	* bio7
8	* Mean Temperature of Wettest Quarter	* bio8
9	* Mean Temperature of Driest Quarter	* bio9
10	Mean Temperature of Warmest Quarter	bio10
11	Mean Temperature of Coldest Quarter	bio11
12	Annual Precipitation	bio12
13	Precipitation of Wettest Month	bio13
14	Precipitation of Driest Month	bio14
15	* Precipitation Seasonality (Coefficient of Variation)	* bio15
16	Precipitation of Wettest Quarter	bio16
17	* Precipitation of Driest Quarter	* bio17
18	* Precipitation of Warmest Quarter	* bio18
19	* Precipitation of Coldest Quarter	* bio19

**Table 2:** Area Under the Curve (AUC) values for training and test data, along with the 10th percentile training presence threshold, fractional predicted area, and True Skill Statistics (TSS) values for three bumblebee species.

Species	AUC Training Data	AUC Test Data	10th Percentile Value	Fractional Predicted Area	TSS
*B. haemorrhoidalis*	0.99	0.97	0.282	0.021	0.55
* B. rufofaciatus*	0.98	0.99	0.147	0.037	0.64
*B. subtypicus*	0.97	0.99	0.411	0.068	0.86

**Table 3:** Percentage overlap of suitable areas for all three bumblebee species in north part of Pakistan at current and future scenarios.

Sr. No.	Cities	Percentage of Suitable Area				
		Current	SSP1 (2021–2040)	SSP1 (2041–2060)	SSP4 (2021–2040)	SSP4 (2041–2060)
1	Islamabad	100	0	0	0	0
2	Mardan	100	0.04	0	0.09	0
3	Poonch	100	4.71	5	20.71	32.09
4	Sudhnati	100	0.29	0	7.37	10.69
5	Swabi	100	0	0	0	0
6	Haripur	99.89	0	6.25	0	0
7	Buner	99.48	1.36	0	2.08	1.28
8	Orakzai	98.87	25.68	0	14.19	4.40
9	Rawalpindi	98.33	0.00	0.88	0.00	0
10	Malakand P.A.	98.19	0.74	0	1.14	0.20
11	Kotli	95.26	0	0	0.30	0.22
12	Charsadda	94.97	0	0	0	0
13	Bagh	93.48	19.18	20.69	31.13	47.97
14	Abbottabad	91.69	0	60.98	0	2.63
15	Kurram	89.39	16.50	0	17.32	4.67
16	Shangla	88.59	25.92	2.44	29.56	15.17
17	Muzaffarabad	87.90	22.17	52.94	36.61	44.88
18	Peshawar	87.32	0	0	0	0
19	Nowshera	85.42	0	0	0	0
20	Hangu	81.56	0	0	0	0
21	Attok	73.36	0	0	0	0
22	Battagram	65.78	3.6	15.15	10.49	1.80
23	Adam Khel	62.48	0	0	0	0
24	Khyber	59.45	24.84	1.72	16.39	1.51
25	Mansehra	58.98	4.43	28.57	6.95	3.52
26	Dir	57.53	4.04	3.92	6.60	3.60
27	Chakwal	52.46	0	0	0	0
28	Bajaur	52.09	0	0	0	0.71
29	Swat	46.76	23.06	3.74	31.44	20.75
30	Neelum	27.97	6.05	3.30	8.40	3.43
31	Bhimber	23.82	0	0	0	0
32	Kohistan	19.19	9.1	0	10.26	0.04
33	Mirpur	18.76	0	0	0	0
34	Kohat	17.95	0	0	0	0
35	Karak	13.81	0	0	0	0
36	Jhelum	8.89	0	0	0	0
37	Mohmand	8.15	0	0	0	0.27
38	Kupwara (Gilgit Wazarat)	4.55	0	0	0	0
39	Chilas	3.07	0	0	0	0
40	Gilgit (Tribal Territory)	0.88	0	0	0	0
41	Kargil	0.45	0	0	0	0
42	Gilgit	0.24	0	0	0	0
43	Chitral	0.04	0	1.73	0	0

**Table 4:** Distribution of habitat suitability areas across different suitability classes for *Bombus haemorrhoidalis* in north part of Pakistan.

Cities	Area (km^2^) Distribution in Different Habitat Suitability Classes				
	Very Low	Low	Medium	High	Very High
Abbottabad	10	295	373	1082	1822
Adam Khel	510	551	369	59	0
Attok	10	3201	3919	5065	413
Bagh	120	401	318	557	1405
Bajaur	130	820	1359	352	0
Bannu	2036	393	0	0	0
Battagram	920	424	602	813	165
Bhimber	4	2130	500	0	0
Buner	205	972	2148	0	0
Chakwal	2904	4460	5354	724	0
Charsadda	225	572	1078	0	0
Chilas	8715	2271	48	0	0
Chitral	28,451	569	16	0	0
Dir	2470	1057	2234	2210	2624
Gilgit	58,134	312	8	0	0
Gilgit (Tribal Territory)	6816	789	81	0	0
Hangu	160	386	526	1515	0
Haripur	23	461	3242	0	0
Islamabad	7	1660	0	0	0
Jhelum	1806	2318	2333	358	0
Karak	83	4001	1290	530	69
Kargil	29,764	127	0	0	0
Khyber	424	1177	1463	2014	397
Kohat	3	4475	482	182	81
Kohistan	9055	2161	1197	910	837
Kotli	1966	1694	0	0	0
Kupwara (Gilgit Wazarat)	7648	397	49	0	0
Kurram	336	478	988	2315	2325
Ladakh (Leh)	22,369	0	0	0	0
Malakand P.A.	58	733	1153	0	0
Mansehra	3821	910	774	2053	2244
Mardan	603	1615	902	0	0
Mirpur	276	1310	209	0	0
Mohmand	112	2860	1169	288	44
Muzaffarabad	148	541	442	870	1792
Neelum	7416	1768	608	321	160
Nowshera	632	2542	420	72	0
Orakzai	7	144	1458	1060	0
Peshawar	348	380	317	1475	0
Poonch	1	391	1181	0	0
Rawalpindi	8	430	3502	6373	0
Shangla	153	170	733	1499	639
Sudhnati	365	497	0	0	0
Swabi	225	2137	722	0	0
Swat	5066	769	716	1272	2671

**Table 5:** Distribution of habitat suitability areas across different suitability classes for *Bombus rufofasciatus* in north part of Pakistan.

Cities	Area (km^2^) Distribution in Different Habitat Suitability Classes				
	Very Low	Low	Medium	High	Very High
Abbottabad	400	3182	0	0	0
Adam Khel	278	943	268	0	0
Attok	20	3389	6396	2771	31
Bagh	290	1115	1396	0	0
Bajaur	61	1427	1033	140	0
Bannu	171	2258	0	0	0
Battagram	42	83	131	1577	1090
Bhimber	2213	421	0	0	0
Buner	72	1956	1297	0	0
Chakwal	6797	6645	0	0	0
Charsadda	222	188	198	1268	0
Chilas	3725	1749	2350	2264	945
Chitral	23,950	3984	870	209	23
Dir	743	820	2795	4327	1911
Gilgit	51,088	5494	1424	427	21
Gilgit (Tribal Territory)	3943	1537	1530	644	30
Hangu	271	718	1553	44	0
Haripur	4	1871	1850	0	0
Islamabad	1491	177	0	0	0
Jhelum	6322	492	0	0	0
Karak	86	5031	808	48	0
Kargil	24,857	3382	1231	324	98
Khyber	1087	892	1005	486	2005
Kohat	4324	700	160	40	0
Kohistan	4101	1612	1710	4013	2723
Kotli	184	3010	456	10	0
Kupwara (Gilgit Wazarat)	5135	1041	892	619	407
Kurram	136	294	1659	4352	0
Ladakh (Leh)	22,222	147	0	0	0
Malakand P.A.	79	985	603	277	0
Mansehra	1331	1057	534	4545	2336
Mardan	1	808	2310	0	0
Mirpur	1	1480	314	0	0
Mohmand	3187	1111	154	21	0
Muzaffarabad	4	83	2178	1529	0
Neelum	3442	1487	640	608	4095
Nowshera	430	1680	762	794	0
Orakzai	1	89	804	1775	0
Peshawar	229	818	626	846	0
Poonch	343	808	423	0	0
Rawalpindi	146	9202	965	0	0
Shangla	11	33	133	2428	589
Sudhnati	1	396	442	23	0
Swabi	471	1460	1153	0	0
Swat	2652	855	554	1696	4737

**Table 6:** Distribution of habitat suitability areas across different suitability classes for *Bombus subtypicus* in north part of Pakistan.

Cities	Area (km2) Distribution in Different Habitat Suitability Classes				
	Very Low	Low	Medium	High	Very High
Abbottabad	14	1005	2563	0	0
Adam Khel	574	916	0	0	0
Attok	3	2313	10,291	0	0
Bagh	11	1063	1727	0	0
Bajaur	1	230	1352	1077	0
Bannu	2083	346	0	0	0
Battagram	25	179	663	1147	909
Bhimber	1088	1543	3	0	0
Buner	274	3051	0	0	0
Chakwal	21	5142	8278	0	0
Charsadda	1875	0	0	0	0
Chilas	1764	2725	4740	1610	196
Chitral	17,324	6636	4020	969	86
Dir	961	1071	1574	3763	3226
Gilgit	32,252	14,490	9380	1872	459
Gilgit (Tribal Territory)	2722	3185	1340	393	45
Hangu	813	1773	0	0	0
Haripur	396	3329	0	0	0
Islamabad	188	1480	0	0	0
Jhelum	266	6483	65	0	0
Karak	575	4442	957	0	0
Kargil	10,211	8191	7781	2504	1204
Khyber	55	605	3734	1081	0
Kohat	3165	2058	0	0	0
Kohistan	3880	3646	3979	1713	941
Kotli	725	2935	0	0	0
Kupwara (Gilgit Wazarat)	1946	2579	1864	1294	411
Kurram	7	151	3201	3082	0
Ladakh (Leh)	15,440	2903	3126	769	131
Malakand P.A.	141	1803	0	0	0
Mansehra	783	1600	1080	1831	4509
Mardan	1	3119	0	0	0
Mirpur	196	1574	24	0	0
Mohmand	7	1142	2476	848	0
Muzaffarabad	10	164	1443	2178	0
Neelum	1157	2846	2084	3051	1133
Nowshera	252	3414	0	0	0
Orakzai	6	1498	1166	0	0
Peshawar	213	2306	0	0	0
Poonch	349	1225	0	0	0
Rawalpindi	6	1901	8407	0	0
Shangla	16	85	208	971	1915
Sudhnati	287	575	0	0	0
Swabi	25	3058	0	0	0
Swat	2715	1571	1287	1641	3280

## Data Availability

All the data generated or analyzed during this study are included in this published article and its Supplementary Materials.

## Supplementary Materials

The following supporting information can be downloaded at https://www.mdpi.com/article/10.3390/insects16010001/s1, Table S1: Distribution of habitat suitability areas across different suitability classes for *Bombus haemorrhoidalis* during SSP1 (2021–2040); Table S2: Distribution of habitat suitability areas across different suitability classes for *Bombus rufofasciatus* during SSP1 (2021–2040); Table S3: Distribution of habitat suitability areas across different suitability classes for *Bombus subtypicus* during SSP1 (2021–2040); Table S4: Distribution of habitat suitability areas across different suitability classes for *Bombus haemorrhoidalis* during SSP1 (2041–2060); Table S5: Distribution of habitat suitability areas across different suitability classes for *Bombus rufofasciatus* during SSP1 (2041–2060); Table S6: Distribution of habitat suitability areas across different suitability classes for *Bombus subtypicus* during SSP1 (2041–2060); Table S7: Distribution of habitat suitability areas across different suitability classes for *Bombus haemorrhoidalis* during SSP4 (2021–2040); Table S8: Distribution of habitat suitability areas across different suitability classes for *Bombus rufofasciatus* during SSP4 (2021–2040); Table S9: Distribution of habitat suitability areas across different suitability classes for *Bombus subtypicus* during SSP4 (2021–2040); Table S10: Distribution of habitat suitability areas across different suitability classes for *Bombus haemorrhoidalis* during SSP4 (2041–2060); Table S11: Distribution of habitat suitability areas across different suitability classes for *Bombus rufofasciatus* during SSP4 (2041–2060); Table S12: Distribution of habitat suitability areas across different suitability classes for *Bombus typicus* during SSP4 (2041–2060); Figure S1: Contribution of bioclimatic factors in the spatial distribution of the three most important bumblebee species in northern areas of Pakistan under future scenarios.
