# Supplementary material for: Temperature-Related Bioclimatic Variables Play a Greater Role in the Spatial Distribution of Bumblebee Species in Northern Pakistan
Source: Insects. 2024 Dec 24;16(1):1. doi: 10.3390/insects16010001 (PMC11765756; doi:10.3390/insects16010001)
Supplement: Supplementary file 1 [file insects-16-00001-s001.zip › insects-3344543-supplementary.pdf]

insects

MDPI

Supplementary Materials

Table S1. Distribution of habitat suitability areas across different suitability classes for *Bombus haemorrhoidalis* during SSP1 (2021–2040).

Cities	Area (km²) distribution in different habitat suitability classes				
	Very Low	Low	Medium	High	Very high
Abbottabad	62	1105	2,415	0	0
Adam Khel	14	1015	449	11	0
Attok	396	3,609	3,348	5,254	0
Bagh	130	821	646	640	594
Bajaur	2,600	58	3	0	0
Bannu	2,207	222	0	0	0
Battagram	649	322	237	496	1,220
Bhimber	25	2,575	64	0	0
Buner	223	3,102	0	0	0
Chakwal	76	1,663	5,794	3,483	2,425
Charsadda	1,207	640	28	0	0
Chilas	10,818	216	0	0	0
Chitral	29,003	49	0	0	0
Dir	2,852	4,923	1,855	776	189
Gilgit	58,518	0	0	0	0
Gilgit (Tribal Territory)	7,582	103	0	0	0
Hangu	98	684	1,280	524	0
Haripur	1,701	2,024	0	0	0
Islamabad	1,444	223	0	0	0
Jhelum	933	1,102	589	3,000	1,190
Karak	25	1,485	2,240	1,535	688
Kargil	29,964	0	0	0	0
Khyber	834	2,548	991	1,099	6
Kohat	319	4,683	219	1	0
Kohistan	9,715	2,152	981	695	616
Kotli	165	2,435	1,084	0	0
Kupwara (Gilgit Wazarat)	8,089	6	0	0	0
Kurram	490	678	3,058	1,426	801
Ladakh (Leh)	22,534	0	0	0	0
Malakand P.A.	496	832	332	284	0
Mansehra	3,811	408	338	2,641	2,604
Mardan	191	1,576	1,354	0	0
Mirpur	40	1,456	300	0	0
Mohmand	565	3,888	25	0	0
Muzaffarabad	71	472	664	1,164	1,453
Neelum	8,733	837	520	254	55
Nowshera	48	1,658	1,855	105	0
Orakzai	175	476	1,246	772	0
Peshawar	76	1,898	545	0	0
Poonch	229	427	479	454	0
Rawalpindi	239	4,344	5,730	0	0
Shangla	153	57	81	495	2,409

Sudhnati	47	444	372	0	0
Swabi	133	2,951	0	0	0
Swat	5,462	647	582	1,050	2,753

Table S2. Distribution of habitat suitability areas across different suitability classes for *Bombus rufofasciatus* during SSP1 (2021–2040).

Cities	Area (km²) distribution in different habitat suitability classes				
	Very Low	Low	Medium	High	Very high
Abbottabad	3208	281	89	4	0
Adam Khel	1465	24	0	0	0
Attok	12607	0	0	0	0
Bagh	622	312	526	765	606
Bajaur	2395	201	57	8	0
Bannu	2429	0	0	0	0
Battagram	1341	304	287	386	606
Bhimber	2664	0	0	0	0
Buner	3273	18	10	24	0
Chakwal	13442	0	0	0	0
Charsadda	1875	0	0	0	0
Chilas	1870	2005	2066	2330	2763
Chitral	8104	9947	7142	3564	295
Dir	3233	1793	1272	2546	1751
Gilgit	33394	15040	9344	683	58
Gilgit (Tribal Territory)	349	1290	2849	1937	1259
Hangu	2586	0	0	0	0
Haripur	3725	0	0	0	0
Islamabad	1668	0	0	0	0
Jhelum	6814	0	0	0	0
Karak	5973	0	0	0	0
Kargil	26371	3266	314	14	0
Khyber	2826	360	353	927	1010
Kohat	5213	8	1	0	0
Kohistan	557	961	3351	4042	5250
Kotli	3684	0	0	0	0
Kupwara (Gilgit Wazarat)	2186	2973	1980	897	58
Kurram	2491	497	760	1559	1146
Ladakh (Leh)	22534	0	0	0	0
Malakand P.A.	1659	273	11	1	0
Mansehra	4458	496	1169	2011	1669
Mardan	3110	8	1	0	0
Mirpur	1795	0	0	0	0
Mohmand	4385	79	13	1	0
Muzaffarabad	1276	533	748	815	452
Neelum	189	1973	2473	2504	3260
Nowshera	3666	0	0	0	0
Orakzai	1383	253	304	598	131
Peshawar	2520	0	0	0	0
Poonch	838	146	246	335	24
Rawalpindi	10288	25	0	0	0
Shangla	2060	81	8	465	579

Sudhnati	820	37	6	0	0
Swabi	3083	0	0	0	0
Swat	418	1108	2155	3637	3175

Table S3. Distribution of habitat suitability areas across different suitability classes for *Bombus subtypicus* during SSP1 (2021–2040).

Cities	Area (km²) distribution in different habitat suitability classes				
	Very Low	Low	Medium	High	Very high
Abbottabad	31	2113	1439	0	0
Adam Khel	1429	61	0	0	0
Attok	3866	8643	98	0	0
Bagh	158	304	2026	342	0
Bajaur	637	1611	413	0	0
Bannu	2429	0	0	0	0
Battagram	800	1477	647	0	0
Bhimber	2664	0	0	0	0
Buner	647	2400	278	0	0
Chakwal	13378	64	0	0	0
Charsadda	415	1460	0	0	0
Chilas	7	1364	4453	5210	0
Chitral	875	8338	11251	8589	0
Dir	1689	8793	114	0	0
Gilgit	827	5241	11107	41343	0
Gilgit (Tribal Territory)	130	3061	4494	0	0
Hangu	2224	362	0	0	0
Haripur	1491	2186	48	0	0
Islamabad	1651	17	0	0	0
Jhelum	6814	0	0	0	0
Karak	1210	4764	0	0	0
Kargil	4	1309	6529	22123	0
Khyber	280	1959	3239	0	0
Kohat	148	3055	2009	10	0
Kohistan	89	4369	9701	0	0
Kotli	1864	1071	594	155	0
Kupwara (Gilgit Wazarat)	244	1351	6499	0	0
Kurram	232	2767	3455	0	0
Ladakh (Leh)	1238	2510	3680	4887	10220
Malakand P.A.	66	1878	0	0	0
Mansehra	1855	2343	1505	4100	0
Mardan	2768	352	0	0	0
Mirpur	1795	0	0	0	0
Mohmand	1236	2578	664	0	0
Muzaffarabad	287	707	2743	88	0
Neelum	1768	8631	0	0	0
Nowshera	346	3293	27	0	0
Orakzai	17	1272	1381	0	0
Peshawar	640	1879	0	0	0
Poonch	3	181	350	1054	0
Rawalpindi	8855	906	553	0	0
Shangla	226	1143	1686	138	0

Sudhnati	76	259	284	243	0
Swabi	4	2007	1073	0	0
Swat	7094	3400	0	0	0

Table S4. Distribution of habitat suitability areas across different suitability classes for *Bombus haemorrhoidalis* during SSP1 (2041–2060).

Cities	Area (km²) distribution in different habitat suitability classes				
	Very Low	Low	Medium	High	Very high
Abbottabad	88	893	2602	0	0
Adam Khel	198	1289	3	0	0
Attok	346	6332	5816	0	112
Bagh	85	228	284	1618	616
Bajaur	1395	1102	137	0	27
Bannu	1268	1010	151	0	0
Battagram	1293	410	379	472	370
Bhimber	89	2097	478	0	0
Buner	415	2414	496	0	0
Chakwal	44	2935	7281	0	3182
Charsadda	637	1228	10	0	0
Chilas	10608	266	107	13	40
Chitral	28362	594	90	0	7
Dir	3558	3037	3464	8	529
Gilgit	56934	906	431	79	168
Gilgit (Tribal Territory)	7679	6	0	0	0
Hangu	247	2183	155	0	0
Haripur	106	2025	1420	0	174
Islamabad	550	1118	0	0	0
Jhelum	157	3476	3181	0	0
Karak	632	4757	585	0	0
Kargil	29256	586	68	18	35
Khyber	377	3182	1612	1	304
Kohat	2698	2494	31	0	0
Kohistan	13380	557	198	0	25
Kotli	112	2042	1530	0	0
Kupwara (Gilgit Wazarat)	7761	137	79	44	73
Kurram	1128	3074	1814	17	421
Ladakh (Leh)	22534	0	0	0	0
Malakand P.A.	835	1029	81	0	0
Mansehra	4408	389	630	3225	1152
Mardan	1326	1764	31	0	0
Mirpur	887	907	0	0	0
Mohmand	3	2445	1971	0	59
Muzaffarabad	124	246	454	2107	893
Neelum	9715	302	218	35	129
Nowshera	21	3308	331	0	6
Orakzai	47	1591	677	0	355
Peshawar	2503	17	0	0	0
Poonch	1	387	1200	0	0
Rawalpindi	129	3148	6318	0	718
Shangla	305	249	814	288	1537

Sudhnati	37	349	476	0	0
Swabi	1012	1857	215	0	0
Swat	6222	909	1262	1090	1012

Table S5. Distribution of habitat suitability areas across different suitability classes for *Bombus rufofasciatus* during SSP1 (2041–2060).

Cities	Area (km^2) distribution in different habitat suitability classes				
	Very Low	Low	Medium	High	Very high
Abbottabad	34	0	0	0	0
Adam Khel	16	0	0	0	0
Attok	127	0	0	0	0
Bagh	21	1	0	0	0
Bajaur	27	0	0	0	0
Bannu	24	0	0	0	0
Battagram	21	1	4	0	0
Bhimber	28	0	0	0	0
Buner	31	0	0	0	0
Chakwal	131	0	0	0	0
Charsadda	17	0	0	0	0
Chilas	59	21	8	14	3
Chitral	102	55	48	45	41
Dir	58	8	6	10	21
Gilgit	410	96	71	16	0
Gilgit (Tribal Territory)	37	14	11	14	1
Hangu	28	0	0	0	0
Haripur	37	0	0	0	0
Islamabad	18	0	0	0	0
Jhelum	68	0	0	0	0
Karak	55	0	0	0	0
Kargil	252	33	10	4	0
Khyber	52	1	0	0	0
Kohat	54	0	0	0	0
Kohistan	55	52	20	10	6
Kotli	42	0	0	0	0
Kupwara (Gilgit Wazarat)	51	16	10	4	0
Kurram	62	1	3	1	0
Ladakh (Leh)	222	3	0	0	0
Malakand P.A.	23	0	0	0	0
Mansehra	66	25	10	0	0
Mardan	28	0	0	0	0
Mirpur	16	0	0	0	0
Mohmand	45	0	0	0	0
Muzaffarabad	37	1	0	0	0
Neelum	49	21	13	4	14
Nowshera	38	0	0	0	0
Orakzai	24	0	0	0	0
Peshawar	25	0	0	0	0
Poonch	16	0	0	0	0
Rawalpindi	103	0	0	0	0
Shangla	35	0	0	0	0

Sudhnati	8	0	0	0	0
Swabi	33	0	0	0	0
Swat	54	8	6	11	25

Table S6. Distribution of habitat suitability areas across different suitability classes for *Bombus subtypicus* during SSP1 (2041–2060).

Cities	Area (km²) distribution in different habitat suitability classes				
	Very Low	Low	Medium	High	Very high
Abbottabad	397	3185	0	0	0
Adam Khel	123	1365	1	0	0
Attok	6804	5778	24	0	0
Bagh	721	844	1266	0	0
Bajaur	4	2619	38	0	0
Bannu	2429	0	0	0	0
Battagram	1080	1844	0	0	0
Bhimber	1208	1456	0	0	0
Buner	418	2907	0	0	0
Chakwal	13302	140	0	0	0
Charsadda	1788	88	0	0	0
Chilas	668	6209	4156	0	0
Chitral	3397	7615	7477	5985	4579
Dir	1399	7981	1215	0	0
Gilgit	37669	15508	4242	1070	30
Gilgit (Tribal Territory)	71	6881	733	0	0
Hangu	2436	147	3	0	0
Haripur	109	2336	1280	0	0
Islamabad	365	1303	0	0	0
Jhelum	25	3803	2986	0	0
Karak	5973	0	0	0	0
Kargil	18256	10762	789	157	0
Khyber	3540	1430	507	0	0
Kohat	4778	445	0	0	0
Kohistan	1359	7950	4700	150	0
Kotli	950	2734	0	0	0
Kupwara (Gilgit Wazarat)	471	5537	1754	334	0
Kurram	1796	2197	1560	900	0
Ladakh (Leh)	22370	164	0	0	0
Malakand P.A.	1	1837	106	0	0
Mansehra	1170	2905	5727	0	0
Mardan	1403	1608	109	0	0
Mirpur	1170	625	0	0	0
Mohmand	2623	1833	23	0	0
Muzaffarabad	280	1221	2323	0	0
Neelum	1053	4083	3360	1904	0
Nowshera	3666	0	0	0	0
Orakzai	209	1820	632	8	0
Peshawar	2520	0	0	0	0
Poonch	1020	530	38	0	0
Rawalpindi	967	5685	3543	119	0
Shangla	688	2506	0	0	0

Sudhnati	444	410	8	0	0
Swabi	1234	1458	391	0	0
Swat	99	2787	4429	3180	0

Table S7. Distribution of habitat suitability areas across different suitability classes for *Bombus haemorrhoidalis* during SSP4 (2021–2040).

**Cities**	**Area (km²) distribution in different habitat suitability classes**				
	Very Low	Low	Medium	High	Very high
Abbottabad	35	184	1570	1793	0
Adam Khel	246	993	236	14	0
Attok	1491	4129	6711	276	0
Bagh	321	695	521	596	697
Bajaur	900	1731	28	1	0
Bannu	2264	165	0	0	0
Battagram	783	414	232	715	780
Bhimber	1648	1001	16	0	0
Buner	1937	1388	0	0	0
Chakwal	16	1615	4076	7736	0
Charsadda	23	1109	743	0	0
Chilas	10647	387	0	0	0
Chitral	28869	184	0	0	0
Dir	2976	5339	1367	557	358
Gilgit	58518	0	0	0	0
Gilgit (Tribal Territory)	7665	20	0	0	0
Hangu	423	944	1112	107	0
Haripur	3245	480	0	0	0
Islamabad	239	1391	38	0	0
Jhelum	259	1376	1834	3140	205
Karak	79	2134	2603	1157	0
Kargil	29964	0	0	0	0
Khyber	1458	1875	554	553	1037
Kohat	4111	1051	57	4	0
Kohistan	9861	2099	805	486	909
Kotli	95	2887	702	0	0
Kupwara (Gilgit Wazarat)	8087	7	0	0	0
Kurram	441	1617	1867	1143	1386
Ladakh (Leh)	22534	0	0	0	0
Malakand P.A.	708	516	350	370	0
Mansehra	3815	574	572	3107	1734
Mardan	514	1330	1276	0	0
Mirpur	1094	700	1	0	0
Mohmand	3376	1078	20	4	0
Muzaffarabad	215	635	517	1010	1447
Neelum	8562	1002	462	273	100
Nowshera	513	2074	1033	45	0
Orakzai	178	520	824	1147	0
Peshawar	2513	7	0	0	0
Poonch	114	386	520	568	0
Rawalpindi	3558	4644	2111	0	0
Shangla	141	62	76	1029	1885

Sudhnati	7	423	432	0	0
Swabi	1706	1378	0	0	0
Swat	5405	606	393	657	3433

Table S8. Distribution of habitat suitability areas across different suitability classes for *Bombus rufofasciatus* during SSP4 (2021–2040).

Cities	Area (km²) distribution in different habitat suitability classes				
	Very Low	Low	Medium	High	Very high
Abbottabad	3107	345	126	4	0
Adam Khel	1393	92	4	0	0
Attok	12607	0	0	0	0
Bagh	424	226	531	562	1087
Bajaur	2288	283	79	11	0
Bannu	2429	0	0	0	0
Battagram	1033	261	212	424	993
Bhimber	2664	0	0	0	0
Buner	3245	17	27	37	0
Chakwal	13442	0	0	0	0
Charsadda	1875	0	0	0	0
Chilas	1567	2760	2660	2452	1595
Chitral	12686	10986	4581	757	42
Dir	2367	2333	1967	2466	1463
Gilgit	38857	17972	1515	174	0
Gilgit (Tribal Territory)	293	2626	2870	1885	11
Hangu	2586	0	0	0	0
Haripur	3725	0	0	0	0
Islamabad	1668	0	0	0	0
Jhelum	6814	0	0	0	0
Karak	5973	0	0	0	0
Kargil	28374	1545	34	11	0
Khyber	2955	594	616	602	711
Kohat	5210	4	8	0	0
Kohistan	479	653	3526	4827	4675
Kotli	3656	11	8	8	0
Kupwara (Gilgit Wazarat)	2226	4083	1535	252	0
Kurram	2299	1374	609	1246	926
Ladakh (Leh)	22534	0	0	0	0
Malakand P.A.	1584	304	55	1	0
Mansehra	4078	564	588	2537	2036
Mardan	3083	25	11	0	0
Mirpur	1795	0	0	0	0
Mohmand	4362	76	34	6	0
Muzaffarabad	965	253	759	904	943
Neelum	137	2001	2251	2193	3817
Nowshera	3640	25	0	0	0
Orakzai	1273	584	374	386	52
Peshawar	2520	0	0	0	0
Poonch	618	133	268	403	167
Rawalpindi	10265	48	0	0	0
Shangla	1918	98	59	659	461

Sudhnati	653	81	24	105	0
Swabi	3083	0	0	0	0
Swat	198	544	3403	3348	3002

Table S9. Distribution of habitat suitability areas across different suitability classes for *Bombus subtypicus* during SSP4 (2021–2040).

**Cities**	**Area (km²) distribution in different habitat suitability classes**				
	Very Low	Low	Medium	High	Very high
Abbottabad	1464	2118	0	0	0
Adam Khel	1126	363	0	0	0
Attok	356	7713	4538	0	0
Bagh	86	610	2131	3	0
Bajaur	842	1218	588	13	0
Bannu	1407	1022	0	0	0
Battagram	321	2432	171	0	0
Bhimber	2645	18	0	0	0
Buner	1498	1827	0	0	0
Chakwal	4834	8607	0	0	0
Charsadda	1618	257	0	0	0
Chilas	1	17	2781	5101	3133
Chitral	4338	11153	10399	3163	0
Dir	4379	5166	1050	0	0
Gilgit	1	1744	7340	17838	31595
Gilgit (Tribal Territory)	1171	4704	1809	0	0
Hangu	910	1676	0	0	0
Haripur	98	3303	325	0	0
Islamabad	1396	271	0	0	0
Jhelum	6814	0	0	0	0
Karak	10	5646	318	0	0
Kargil	236	2084	7565	20079	0
Khyber	485	3907	1085	0	0
Kohat	961	4227	35	0	0
Kohistan	11548	2611	0	0	0
Kotli	762	1939	687	297	0
Kupwara (Gilgit Wazarat)	374	2361	5359	0	0
Kurram	298	3659	2497	0	0
Ladakh (Leh)	2124	3057	3864	4125	9365
Malakand P.A.	59	858	1027	0	0
Mansehra	79	3674	2613	3437	0
Mardan	7	2860	253	0	0
Mirpur	1795	0	0	0	0
Mohmand	1203	2028	1225	23	0
Muzaffarabad	4	985	2835	0	0
Neelum	134	4350	5915	0	0
Nowshera	1251	2415	0	0	0
Orakzai	2518	151	0	0	0
Peshawar	2520	0	0	0	0
Poonch	119	352	1118	0	0
Rawalpindi	7422	2229	663	0	0
Shangla	602	2592	0	0	0

Sudhnati	3	246	326	287	0
Swabi	273	2332	479	0	0
Swat	20	8769	1706	0	0

Table S10. Distribution of habitat suitability areas across different suitability classes for *Bombus haemorrhoidalis* during SSP4 (2041–2060).

Cities	Area (km²) distribution in different habitat suitability classes				
	Very Low	Low	Medium	High	Very high
Abbottabad	105	1270	2207	0	0
Adam Khel	205	1282	3	0	0
Attok	358	6585	5589	75	0
Bagh	85	233	295	878	1340
Bajaur	1460	1053	130	18	0
Bannu	1275	1017	137	0	0
Battagram	1294	421	410	417	382
Bhimber	144	2400	120	0	0
Buner	455	2459	411	0	0
Chakwal	44	3068	7611	2719	0
Charsadda	668	1204	3	0	0
Chilas	10611	267	107	40	8
Chitral	28365	594	90	4	0
Dir	3561	3157	3461	414	3
Gilgit	56938	917	438	163	62
Gilgit (Tribal Territory)	7679	6	0	0	0
Hangu	257	2205	124	0	0
Haripur	119	2206	1277	123	0
Islamabad	643	1025	0	0	0
Jhelum	205	3725	2884	0	0
Karak	644	4778	551	0	0
Kargil	29262	585	69	34	14
Khyber	382	3247	1627	222	0
Kohat	2719	2474	30	0	0
Kohistan	13382	562	201	14	0
Kotli	168	2497	1019	0	0
Kupwara (Gilgit Wazarat)	7761	137	82	81	34
Kurram	1133	3137	1802	382	0
Ladakh (Leh)	22534	0	0	0	0
Malakand P.A.	863	1058	23	0	0
Mansehra	4408	397	711	1259	3028
Mardan	1461	1655	4	0	0
Mirpur	1005	790	0	0	0
Mohmand	3	2535	1892	48	0
Muzaffarabad	124	253	513	993	1940
Neelum	9718	309	228	119	25
Nowshera	23	3346	295	1	0
Orakzai	47	1614	742	267	0
Peshawar	2508	11	0	0	0
Poonch	4	451	1133	0	0
Rawalpindi	148	3522	6385	259	0
Shangla	305	266	924	1537	161

Sudhnati	51	401	410	0	0
Swabi	1088	1855	140	0	0
Swat	6223	948	1297	1180	845

Table S11. Distribution of habitat suitability areas across different suitability classes for *Bombus rufofasciatus* during SSP4 (2041–2060).

Cities	Area (km²) distribution in different habitat suitability classes				
	Very Low	Low	Medium	High	Very high
Abbottabad	3137	345	99	1	0
Adam Khel	1395	92	3	0	0
Attok	12607	0	0	0	0
Bagh	434	246	596	516	1039
Bajaur	2319	266	68	8	0
Bannu	2429	0	0	0	0
Battagram	1056	261	215	441	951
Bhimber	2664	0	0	0	0
Buner	3246	20	30	30	0
Chakwal	13442	0	0	0	0
Charsadda	1875	0	0	0	0
Chilas	1662	3010	2596	2343	1423
Chitral	13679	10933	3813	596	31
Dir	2554	2400	2096	2173	1374
Gilgit	39675	17749	975	119	0
Gilgit (Tribal Territory)	317	3123	2665	1578	1
Hangu	2586	0	0	0	0
Haripur	3725	0	0	0	0
Islamabad	1668	0	0	0	0
Jhelum	6814	0	0	0	0
Karak	5973	0	0	0	0
Kargil	28601	1341	18	4	0
Khyber	3006	602	627	642	601
Kohat	5210	7	6	0	0
Kohistan	490	921	3784	4697	4266
Kotli	3663	4	8	8	0
Kupwara (Gilgit Wazarat)	2490	4112	1369	123	0
Kurram	2361	1413	581	1303	796
Ladakh (Leh)	22534	0	0	0	0
Malakand P.A.	1666	239	40	0	0
Mansehra	4139	531	841	2419	1872
Mardan	3089	24	7	0	0
Mirpur	1795	0	0	0	0
Mohmand	4369	75	28	6	0
Muzaffarabad	967	317	830	856	855
Neelum	202	2148	2359	2017	3674
Nowshera	3640	25	0	0	0
Orakzai	1304	602	352	374	37
Peshawar	2520	0	0	0	0
Poonch	625	133	298	407	126
Rawalpindi	10272	41	0	0	0
Shangla	1928	93	79	670	424

Sudhnati	664	69	37	92	0
Swabi	3083	0	0	0	0
Swat	199	786	3550	3191	2768

Table S12. Distribution of habitat suitability areas across different suitability classes for *Bombus typicus* during SSP4 (2041–2060).

Cities	Area (km²) distribution in different habitat suitability classes				
	Very Low	Low	Medium	High	Very high
Abbottabad	397	3185	0	0	0
Adam Khel	123	1365	1	0	0
Attok	6804	5778	24	0	0
Bagh	721	844	1266	0	0
Bajaur	4	2619	38	0	0
Bannu	2429	0	0	0	0
Battagram	1080	1844	0	0	0
Bhimber	1208	1456	0	0	0
Buner	418	2907	0	0	0
Chakwal	13302	140	0	0	0
Charsadda	1788	88	0	0	0
Chilas	668	6209	4156	0	0
Chitral	3397	7615	7477	5985	4579
Dir	1399	7981	1215	0	0
Gilgit	37669	15508	4242	1070	30
Gilgit (Tribal Territory)	71	6881	733	0	0
Hangu	2436	147	3	0	0
Haripur	109	2336	1280	0	0
Islamabad	365	1303	0	0	0
Jhelum	25	3803	2986	0	0
Karak	5973	0	0	0	0
Kargil	18256	10762	789	157	0
Khyber	3540	1430	507	0	0
Kohat	4778	445	0	0	0
Kohistan	1359	7950	4700	150	0
Kotli	950	2734	0	0	0
Kupwara (Gilgit Wazarat)	471	5537	1754	334	0
Kurram	1796	2197	1560	900	0
Ladakh (Leh)	22370	164	0	0	0
Malakand P.A.	1	1837	106	0	0
Mansehra	1170	2905	5727	0	0
Mardan	1403	1608	109	0	0
Mirpur	1170	625	0	0	0
Mohmand	2623	1833	23	0	0
Muzaffarabad	280	1221	2323	0	0
Neelum	1053	4083	3360	1904	0
Nowshera	3666	0	0	0	0
Orakzai	209	1820	632	8	0
Peshawar	2520	0	0	0	0
Poonch	1020	530	38	0	0
Rawalpindi	967	5685	3543	119	0
Shangla	688	2506	0	0	0

Sudhnati	444	410	8	0	0
Swabi	1234	1458	391	0	0
Swat	99	2787	4429	3180	0

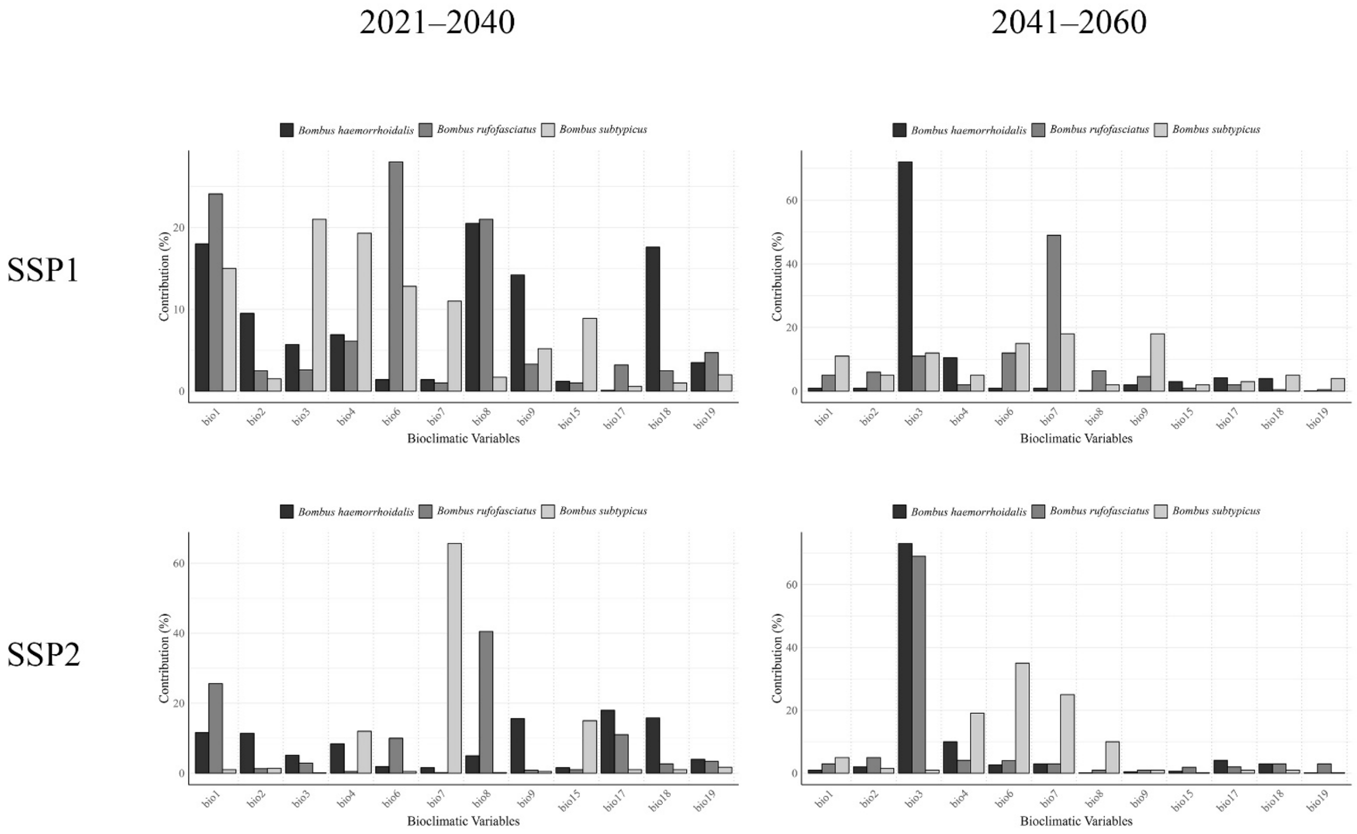

Figure S1. Contribution of bioclimatic factors in the spatial distribution of the three most important bumblebee species in northern areas of Pakistan under future scenarios.
